# Supplementary figures and images for: Metabolic Robustness to Growth Temperature of a Cold- Adapted Marine Bacterium
Source: mSystems. 2023 Feb 27;8(2):e01124-22. doi: 10.1128/msystems.01124-22 (PMC10134870; doi:10.1128/msystems.01124-22)

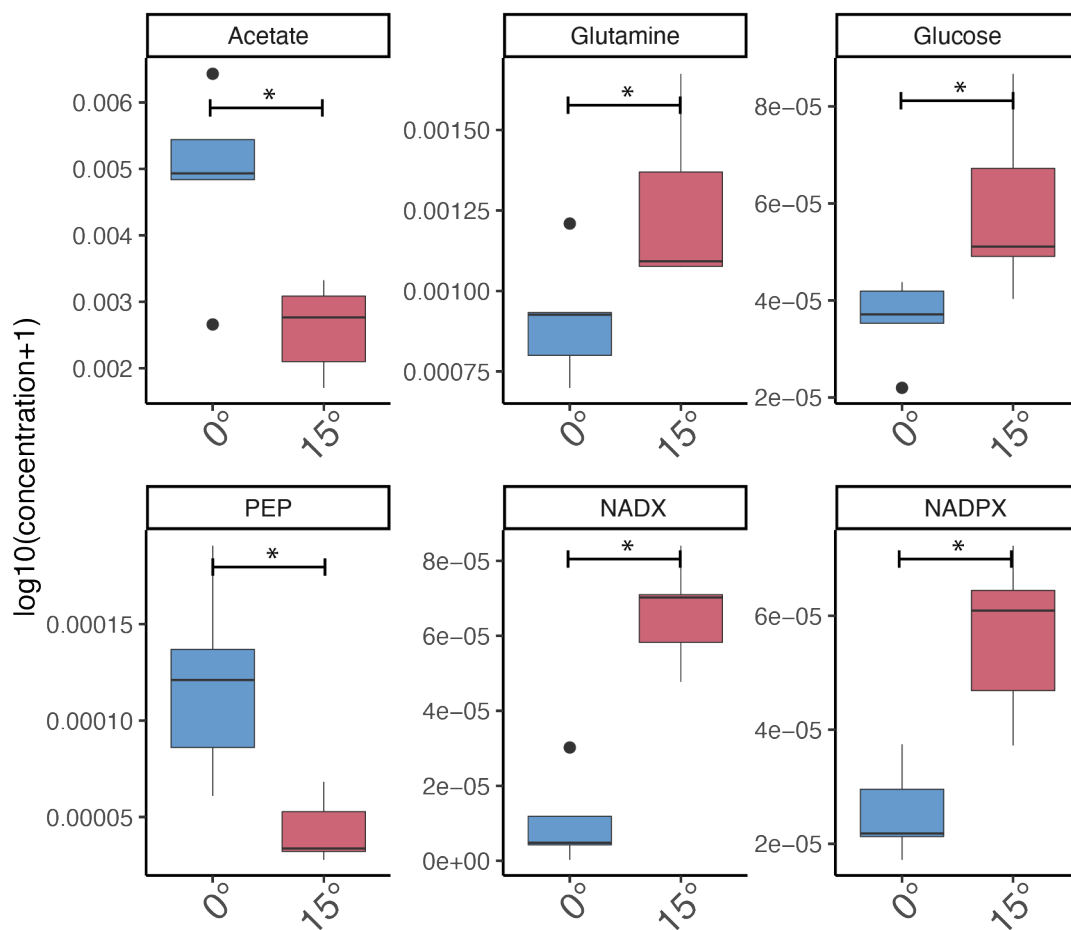

Supplement: FIG S1 [file msystems.01124-22-s0001.pdf]

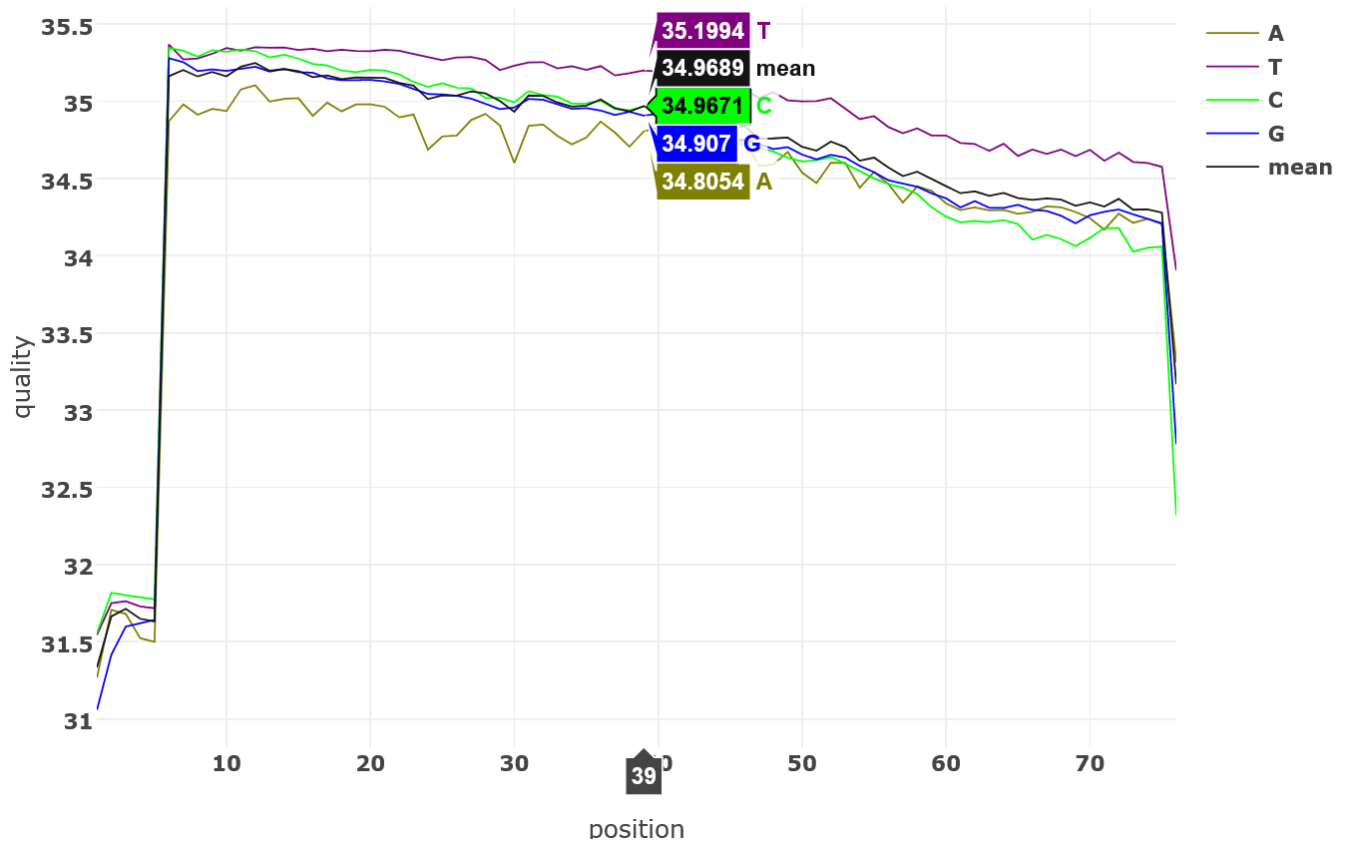

Supplement: FIG S2 [file msystems.01124-22-s0002.pdf]

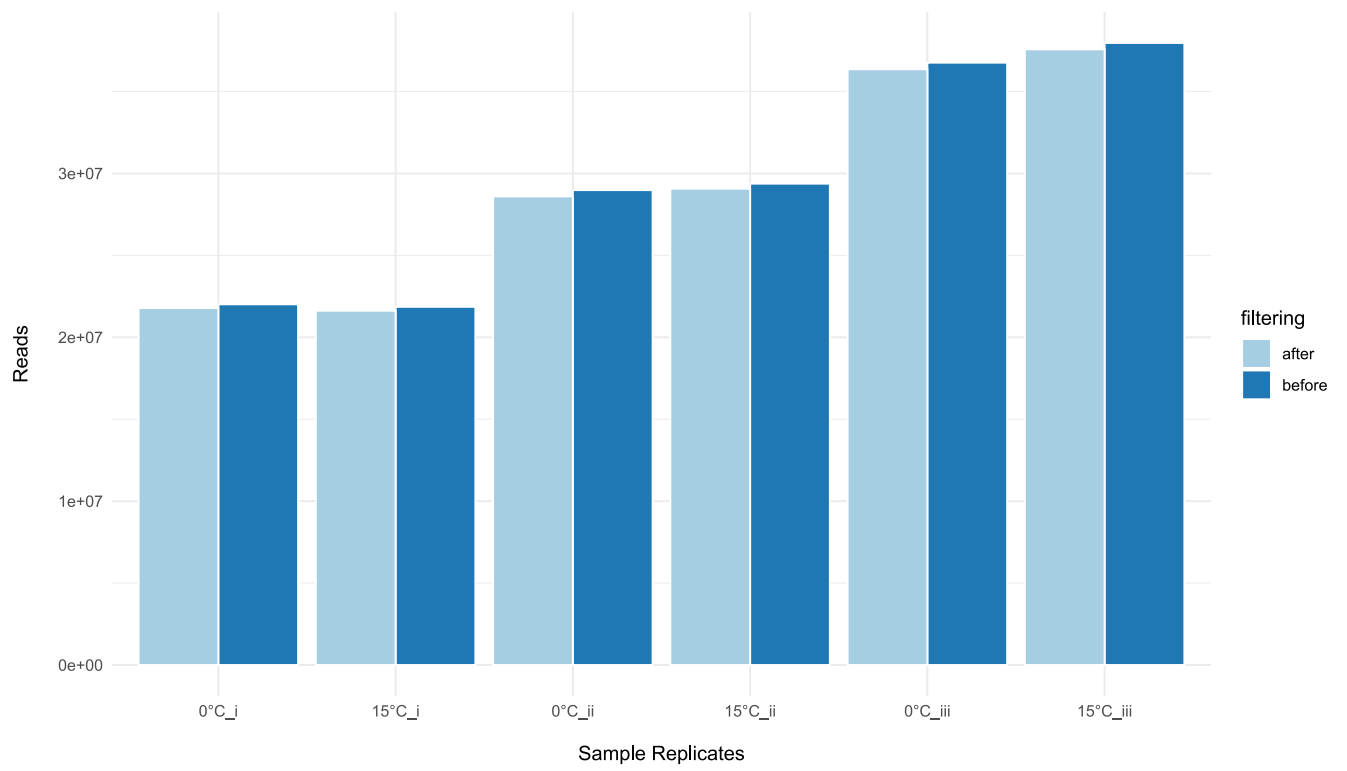

Supplement: FIG S3 [file msystems.01124-22-s0003.pdf]
