# Supplementary material for: Metabolic Robustness to Growth Temperature of a Cold- Adapted Marine Bacterium
Source: mSystems. 2023 Feb 27;8(2):e01124-22. doi: 10.1128/msystems.01124-22 (PMC10134870; doi:10.1128/msystems.01124-22)
Supplement: TABLE S1 [file msystems.01124-22-s0006.pdf]

Accession Number	Number of Reads	Temperature
SRX17780709	18,376,681	15°C
SRX17780711	25,089,797	15°C
SRX17780712	31,526,393	15°C
SRX17780713	17,586,523	0°C
SRX17780714	23,037,026	0°C
SRX17780710	29,173,509	0°C
