# Supplementary material for: Metabolic Robustness to Growth Temperature of a Cold- Adapted Marine Bacterium
Source: mSystems. 2023 Feb 27;8(2):e01124-22. doi: 10.1128/msystems.01124-22 (PMC10134870; doi:10.1128/msystems.01124-22)
Supplement: FIG S4 [file msystems.01124-22-s0004.pdf]

```
                              RPN1            RPN2
Ph RS16715      -MSTTTGSVKWFNEAKGFGFIEQESG-PDVFAHFSAITSDGFKTLAEGQRVQFTVTQGQK    58
Ph RS14630      MSNTTTGTVKFFNEAKGFGFIEQESG-ADVFAHFSAISGDGFKTLAEGQRVQFTVTQGQK    59
Ph RS14635      MSNTTTGSVKWFNEAKGFGFIEQESG-ADVFAHFSAIVSDGFKTLAEGQRVQFTVTQGQK    59
Ph RS14640      MSNTTTGSVKWFNEAKGFGFIEQESG-ADVFAHFSAIVSDGFKTLAEGQRVQFTVTQGQK    59
Eco cspE        -MSKIKGNVKWFNESKGFGFITPEDGSKDVFVHFSAIQTNGFKTLAEGQRVEFEITNGAK    59
                   . .* **:**  *** **   .*  ***.*****  :*:*:* ***.*.* : .* *

Ph RS16715      GPQAENIVCI 68
Ph RS14630      GPQAENIVCI 69
Ph RS14635      GPQAENIVCI 69
Ph RS14640      GPQAENIVCI 69
Eco cspE        GPSAANVIAL 69
                ** * *: .:
```
