# Supplementary material for: Metabolic Robustness to Growth Temperature of a Cold- Adapted Marine Bacterium
Source: mSystems. 2023 Feb 27;8(2):e01124-22. doi: 10.1128/msystems.01124-22 (PMC10134870; doi:10.1128/msystems.01124-22)
Supplement: TABLE S2 [file msystems.01124-22-s0005.pdf]

**Locus tag**	**log_2FC**	**Adj. p-value**	**Annotation**
PSHAa0090	1.1108113	0.00000925	|xerC| site-specific recombinase
PSHAa1218	1.8251366	1.52E-13	|recN| DNA repair protein recN
PSHAa2356	1.0226152	0.0000609	|topB| DNA topoisomerase III
PSHAa2361	1.2276516	0.0000048	DNA topoisomerase III (N terminal part)
PSHAa2873	1.1514558	0.000014	|lexA| transcriptional repressor for SOS response
PSHA_RS17095	1.382628818	0.00000669	|xni| putative exonuclease IX
